# Tiotropium for the Treatment of Asthma: Patient Selection and Perspectives

**DOI:** 10.1155/2018/3464960

**Published:** 2018-01-21

**Authors:** V. Madhu Chari, Robert Andrew McIvor

**Affiliations:** McMaster University, Firestone Institute for Respiratory Health, Hamilton, ON, Canada

## Abstract

Asthma is a chronic disease of airway inflammation with a large global burden. Despite established, guideline-based stepwise therapy, a significant proportion of patients remain symptomatic and poorly controlled. As such, there is a need for additional safe, effective, convenient, and cost-effective therapies that can be broadly applied across a range of asthma phenotypes. Tiotropium is a long-acting muscarinic antagonist (LAMA) that leads to bronchodilation by blocking endogenous acetylcholine receptors in the airways. Tiotropium has long been approved for the treatment of chronic obstructive pulmonary disease, and it has recently been recognized for its safety and efficacy in improving lung function and controlling asthma. Evidence from several Phase III trials in the adult and paediatric population has shown that tiotropium is well tolerated and significantly improves a range of endpoints as an add-on treatment to ICS therapy, regardless of baseline characteristics and clinical phenotypes. Consequently, regulatory authorities worldwide have recently licensed tiotropium as the only LAMA approved for the treatment of asthma. This review provides an overview of safety and efficacy data and discusses the use of tiotropium in patients across the range of asthma severities, ages, and phenotypes.

## 1. Introduction

Asthma is a heterogeneous condition that is usually characterized by chronic airway inflammation and defined by symptoms including wheeze, shortness of breath, chest tightness, and cough that vary with time and intensity, along with variable expiratory airflow limitation [1]. This chronic disease is estimated to have a global prevalence of up to 18%, and it is estimated that 400 million people worldwide will be affected by 2025 [1, 2]. Asthma is the most common chronic childhood disease [3] and continues to impose a substantial burden on healthcare systems, society, and families across developing as well as high-income countries [1]. The broad aims of treating asthma are to (1) maintain normal activity levels via symptom control and (2) minimize exacerbations, medication side effects, and the development of fixed airflow limitation.

The Global Initiative for Asthma (GINA) strategy recommends a stepwise approach to the pharmacologic management of asthma, building on a base of inhaled corticosteroids (ICSs) [1]. Data from the UK estimate that approximately 65% of the patients being treated with at least a combination of an ICS and a long-acting β_2_-agonist (LABA) (i.e., GINA Step 3) remain uncontrolled [4]. This places patients at increased risk of exacerbations, which, along with greater disease severity, is associated with increased costs of asthma care, both direct (related to provision of health services) and indirect (lost productivity) [5]. GINA Step 5 options are limited, and their application can be further constrained by access (bronchial thermoplasty), specific biomarker profiles (biologics) [6], or systemic side effects (oral corticosteroids). Thus, there is an ongoing unmet need for additional cost-effective and safe add-ons to ICS-LABA in the optimization of those with persistent asthma at GINA Steps 3–5.

In asthma, adherence to treatment can be quite poor, ranging from <50% in children to 30–70% in adults; this is disconcerting as poor adherence has been associated with not only worse symptoms and quality of life but also exacerbation frequency and mortality [7]. Controller medications with longer half-lives and once-daily administration are not only more convenient but may also improve treatment adherence. Tiotropium is a once-daily, long-acting muscarinic antagonist (LAMA) that has established effectiveness in chronic obstructive pulmonary disease (COPD), improving lung function, quality of life, and exacerbation frequency [8–10]. Recent and emerging data from the tiotropium clinical program (Phase II and III studies) in recent years are demonstrating that tiotropium may have a role as an effective long-acting bronchodilator in asthma as well. In fact, since 2015, tiotropium add-on therapy has been incorporated into the GINA guidelines in the stepwise management of asthma, starting at Step 4.

This review discusses the use of tiotropium in patients across a range of ages and asthma severities and provides an overview of the safety and efficacy data, patient selection considerations, and future perspectives.

## 2. Mechanism of Tiotropium

The bronchial tree in humans is largely innervated by the parasympathetic cholinergic system, and it is the muscarinic receptor subtypes (M1, M2, and M3) which are the predominant functional acetylcholine receptors expressed in the lung [11]. Through the effector tissues of the airway smooth muscle and mucous glands, cholinergic drive and dysfunction of muscarinic receptors contribute to bronchial smooth muscle contraction, airway hyperresponsiveness, and mucus secretion in airway diseases [12]. Furthermore, acetylcholine is thought to exert proinflammatory effects through chemoattraction and subsequent cytokine release [13]. As such, the utility of muscarinic antagonists is facilitating bronchodilation in a means that is complementary to LABAs [2]. The role of short-acting anticholinergics in asthma was evaluated in the beginning of 1980s, with ipratropium bromide—a nonselective M1-, M2-, and M3-receptor antagonist with a duration of action ranging from 4 to 8 hours—being the most widely studied. A meta-analysis demonstrated that, in the acute setting, the early administration of inhaled anticholinergics to short-acting β2-agonist (SABA) therapy particularly benefits children and adults with moderate-to-severe obstruction and also reduces admission rates by 30% [14]. For chronic asthma, a Cochrane review found that short-acting anticholinergics (including ipratropium bromide, oxitropium bromide, and nebulised atropine methonitrate) provided an incremental benefit in peak flow versus placebo and no added benefit to SABAs; however, the authors acknowledge that some patient subgroups may derive some benefit from short-acting anticholinergics, including those intolerant to SABAs and those with nocturnal asthma, concurrent fixed airway obstruction, and asthma of a longer duration [15].

The LAMA tiotropium more selectively antagonizes M1 and M3 receptor subtypes and does so with an up to 20-fold higher affinity than does ipratropium; this along with its slow dissociation from the M3 receptor in particular confers a half-life of approximately 35 hours and thus permits once-daily dosing [16]. In addition to bronchodilation and reduction of secretions and mucus gland hypertrophy, LAMAs have also demonstrated anti-inflammatory effects (e.g., inhibition of alveolar neutrophil migration and decreased levels of IL-6, TNF-α, and leukotriene B4) in vitro and in bronchoalveolar lavages [16].

## 3. Tiotropium in Asthma: The Evidence Base

A number of Phase II studies initially established the efficacy and safety of tiotropium delivered by a Respimat® Soft Mist inhaler (hereafter referred to as tiotropium Respimat; Boehringer Ingelheim, Ingelheim am Rhein, Germany) in adult patients with symptomatic asthma; all of these were randomized, double-blind, placebo-controlled studies and had treatment durations ranging from 4 to 16 weeks. In a crossover study of 107 patients with uncontrolled, severe asthma on a background treatment of (at minimum) high-dose ICSs and LABAs, the add-on of tiotropium Respimat at 5 μg once daily for 8 weeks significantly improved peak FEV1 (difference from placebo 139 mL, *p* < 0.001); the higher dose of 10 μg once daily did not confer any additional bronchodilator benefit [17]. The role of tiotropium as an add-on therapy to ICSs in moderate asthma was then assessed in a dose-ranging, four-way crossover study comparing once-daily tiotropium Respimat at doses of 5, 2.5, and 1 μg to placebo; patients were not permitted to be on an LABA at the time of enrollment [18]. All doses of tiotropium Respimat were found to improve peak FEV1 at 4 weeks compared to placebo, with the 5 μg dose resulting in the largest adjusted mean difference (188 mL, 95% CI: 140–236 mL). With the abovementioned studies establishing the 5 μg dose of tiotropium Respimat as a safe and effective add-on therapy in moderate-to-severe asthma, Timmer et al. sought to investigate whether this would be affected by dosing regimen, comparing twice-daily tiotropium at 2.5 μg to once-daily tiotropium at 5 μg [19]. After 4 weeks, no significant difference in bronchodilator efficacy was found between either dosing strategy, with both providing improvements in the FEV1 AUC_(0–24 h)_ response versus placebo (158_ _mL for 5_ _μg once daily, 149_ _mL for 2.5_ _μg twice daily; both *p* < 0.01). Both the 5 μg once-daily and 2.5_ _μg twice-daily tiotropium Respimat treatments also had comparable improvements in peak and trough FEV1 recordings, as well as predosing morning and evening PEFs.

A comprehensive Phase III study programme detailed below, and summarized in Table 1 [20], has demonstrated the safety and efficacy of tiotropium in adult patients with mild, moderate, and severe asthma. The majority of the Phase III trials have evaluated tiotropium in the Respimat form, with the Tiotropium Bromide as an Alternative to Increased Inhaled Corticosteroid in Patients Inadequately Controlled on a Lower Dose of Inhaled Corticosteroid study (TALC Study, NCT00565266) being one of the exceptions. In this three-way crossover study, adults with moderate, persistent asthma with confirmed bronchodilator reversibility or hyperresponsiveness received 14-week treatments of doubled ICSs (beclomethasone 160 μg twice daily), tiotropium (18 μg via Spiriva® HandiHaler), or salmeterol (50 μg twice daily) [21]. Here, once-daily tiotropium significantly improved morning and evening PEF (*p* < 0.001), FEV1 (*p*=0.004), and asthma control as determined by the Asthma Control Questionnaire (ACQ; *p* < 0.001) compared to doubling of the ICSs. Tiotropium was found to be noninferior to salmeterol when comparing the effects on PEF, FEV1, asthma control and symptoms, and sputum eosinophils.

The PrimoTinA-asthma® study comprised two replicate, randomized controlled trials (NCT00772538 and NCT00776984) which evaluated the long-term (48 weeks) efficacy and safety of tiotropium Respimat (5 µg) in 912 patients with poorly controlled, severe persistent asthma [22]. Adults enrolled in this study had demonstrated persistent airflow limitation as determined by an FEV1 ≤80% or ≤70% of the FVC postbronchodilator and ≥1 exacerbation requiring systemic glucocorticoids in the past year. For background treatment, patients were on high-dose ICSs (≥800 µg budesonide or equivalent/day) and LABAs at minimum; stable dosing of theophylline, leukotriene modifiers, anti-IgE therapy, and oral glucocorticoids (≤5 mg prednisone/equivalent per day) was permitted. When compared to placebo, treatment with 5 μg of tiotropium led to an improvement in the primary outcomes of peak and trough FEV1 at 24 weeks (*p* ≤ 0.01) and time to first exacerbation (282 days for tiotropium versus 226 days for placebo, *p*=0.03; corresponding to a risk reduction of 21% (HR 0.79)) [22]. Despite the effect of add-on tiotropium on exacerbations as well as a secondary outcome of time to first worsening (181 days for tiotropium versus 315 days for placebo, *p* < 0.001; 31% risk reduction (HR 0.69)), the minimal clinically important difference in ACQ-7 and Asthma Quality of Life Questionnaire (AQLQ) scores was not achieved. The MezzoTinA-asthma® trials (NCT01172808 and NCT01172821) were two randomized, double-blinded parallel group trials which assessed the safety and efficacy of tiotropium (at 5 and 2.5 μg once daily), with salmeterol as an active comparator as add-on therapy to medium-dose ICSs (400–800 μg/day) in 2103 patients with moderate, symptomatic asthma [23]. In the pooled analyses, tiotropium and salmeterol had similar efficacy on lung function as determined by peak and trough FEV1 (*p* < 0.001 versus placebo) as well as ACQ-7 responder rates (odds ratio (OR) 1.32 for 5 μg tiotropium, *p*=0.035; 1.33 for 2.5 μg tiotropium, *p*=0.031; 1.46, *p*=0.0039 for salmeterol; *p* values versus placebo) [23], indicating that tiotropium is a safe and effective alternative to salmeterol in this patient population. Due to low event rates, the secondary outcome of the median time to 1st exacerbation could not be calculated. It should be noted that the MezzoTinA-asthma trials were not powered to assess superiority between the different active treatments (tiotropium 2.5 μg and 5 μg, and salmeterol). The GraziaTinA-asthma® study (NCT01316380) then evaluated tiotropium Respimat at 5 and 2.5 μg in 464 patients with mild-to-moderate persistent asthma on only low-to-medium dosing of ICSs (200–400 μg/day) [24]. At 12 weeks, both doses of tiotropium led to a significant increase in the primary outcome of the mean difference in peak FEV1 (5 μg, 128 mL; 2.5 μg, 159 mL; *p* < 0.001) as well as secondary outcomes of trough FEV1, FEV1 AUC_(0–3 h)_, and PEF (morning and evening) compared to placebo; the trial was not powered to demonstrate differences between the two doses of tiotropium. Across the PrimoTinA-asthma, MezzoTinA-asthma, and GraziaTinA-asthma studies, the rates of adverse events were comparable across all treatment, comparator, and placebo arms.

Recent systematic reviews published by the Cochrane Library further examined the role of LAMAs in three particular capacities: (1) as an add-on to ICSs [25], (2) as an alternative to LABAs as an add-on to ICSs [26], and (3) as an add-on therapy to patients not well controlled on combination therapy with ICS-LABA [27]. While the search strategy of the reviews included LAMAs other than tiotropium (including glycopyrronium and umeclidinium), only studies using tiotropium (both HandiHaler® and Respimat forms) were ultimately included in the analyses. Across four studies with 2277 participants, the addition of tiotropium to ICSs was found to lower the rate of exacerbations requiring oral corticosteroids (OR 0.63, 95% CI: 0.46–0.93), as well as to produce a consistent benefit across a range of lung function measures, when compared to ICSs alone [25]. All-cause severe adverse events and exacerbations requiring admission were rare. Analyses from four studies (a total of 2049 patients) comparing LAMAs to LABAs as add-on therapy to ICSs in asthma found that there was no difference in the rate of exacerbations requiring oral corticosteroids and that the slightly worse quality of life scores (as determined by AQLQ and ACQ) and slightly improved lung function (a mean difference of 50 mL in trough FEV1) conferred by LAMA therapy were of unclear clinical significance [26]. Across three studies with 1197 adult asthma patients on combination ICS-LABA therapy, the addition of tiotropium (studied over 48–52 weeks) led to fewer exacerbations; however the confidence interval did not rule out a lack of difference (OR 0.76, 95% CI: 0.57–1.02, moderate-quality evidence); there was high-quality evidence demonstrating a benefit to lung function with the addition of tiotropium (versus placebo) to ICS-LABA [27]. Overall, these reviews illustrated that tiotropium serves as an effective bronchodilator and a possible means of reducing exacerbations across varying severities of asthma in patients who remain symptomatic on at least ICSs, particularly as add-on therapy to ICS/LABA. This reflects the recommendation in the GINA strategy, where tiotropium is now recommended as an alternative add-on treatment at Steps 4 and 5 in patients ≥ 12 years of age with a history of exacerbations [1].

## 4. Patient Selection and Safety Considerations

Evidence from the comprehensive clinical trial programme with tiotropium has demonstrated it as a well-tolerated treatment improving lung function and asthma control in adults, regardless of severity [22–24]. In general, patients enrolled in this programme were men and women aged 18–75 (with a mean age of 43 to 53) who were diagnosed with asthma before age 40, had no prior smoking history or were ex-smokers with a total less than 10 pack-years, and were symptomatic at screening and randomization (as defined by a mean ACQ-7 score of ≥1.5). In the studies of mild and moderate asthma, the diagnosis of asthma had to be confirmed at screening via demonstration of bronchodilator reversibility. Main exclusion criteria in these trials were a diagnosis of COPD, serious coexisting illness (e.g., recent acute coronary syndrome or hospitalization for cardiac failure, lung diseases other than asthma, and recently treated malignancy), and concurrent use of other anticholinergic bronchodilators.

Subsequent subgroup analyses of these trials have provided further insight into the patient phenotypes that would also benefit from tiotropium as an add-on bronchodilator. Asthma can conceptually be distinguished into T_H_2-asthma (e.g., allergic, eosinophilic, and exercise-induced asthma) and non-T_H_2-asthma (e.g., smoking-related, neutrophilic, and obesity-associated). Preplanned analyses of the PrimoTinA-asthma (severe) and MezzoTinA-asthma (moderate) found that ∼20%/∼15% of severe/moderate patients had elevated IgE (≥430 μg/L) and that ∼10%/∼5% of severe/moderate patients had blood eosinophilia (≥0.6 × 10^9^), respectively; analyses were then performed in subgroups of patients with T_H_2- and non-T_H_2-asthma, the former defined by the presence of both elevated IgE and blood eosinophilia [28, 29]. Independent of the T_H_2-status, the addition of once-daily tiotropium (versus placebo) to at least ICSs in adults with moderate-to-severe symptomatic asthma reduced the risk of severe exacerbation and asthma worsening [29] and, furthermore, improved symptom control based on the ACQ-7 responder rates [28]. The link between obesity and asthma is a complex and significant one: obesity is a major risk factor for the development of asthma (possibly through mechanical and proinflammatory changes), and asthma in obese patients tends to be more severe and less treatment responsive [30]. Furthermore, a distinct non-T_H_2 phenotype of “obesity-related asthma” which is later onset and less corticosteroid responsive has been proposed [31]. As well, the entity of asthma in the elderly (AIE) presents a diagnostic and management challenge due to a number of factors, including but not limited to underdiagnosis, high rates of morbidity and mortality, nuances in airway remodelling and structural changes of the aging lung, altered immune response, exclusion from clinical trials, and more common medication adverse events [32]. In subgroup analyses of data from the replicate PrimoTinA-asthma trials (i.e., severe, persistent asthma), the influence of various baseline characteristics on tiotropium's improvements in lung function, exacerbation rate, and asthma control was examined. Of note, at week 24, tiotropium 5 μg led to improvements in the coprimary endpoints of peak FEV1 and trough FEV1, along with the secondary endpoints of time to first severe exacerbation and first episode of asthma worsening independent of age (stratified into the age groups <40, 40–60, and >60) and BMI (stratified into the groups <20, 20 to <25, 25 to <30, and ≥30 kg/m^2^) [33].

Noneosinophilic asthma represents a large proportion of patients with asthma and falls into the endotype of non-T_H_2 asthma. Increased airway neutrophilia—outside of the acute exacerbation setting where neutrophilis can be the dominant inflammatory cell type—has been associated with more clinically severe asthma, lower pre- and postbronchodilator FEV1, thicker airway walls, and more air trapping [31, 34]. Patients with this phenotype also tend to be less corticosteroid sensitive, and biologic agents targeting mediators of noneosinophilic inflammation (e.g., interleukin 17 and tumor necrosis factor-α) have yet to demonstrate clinical efficacy [35]. To date, no large randomized study has evaluated tiotropium or other LAMAs in this particular phenotype. However, a small study (*N* = 17, no placebo arm) demonstrated that the proportion of neutrophils in induced sputum positively correlated with the improvement in FEV1 conferred by a 4-week treatment of tiotropium in patients with severe asthma; these patients were on a baseline treatment of high-dose ICSs (equivalent of 800–1600 μg budesonide/day), and 23.5% were additionally on maintenance oral corticosteroids [36]. As such, a noneosinophilic sputum profile may predict a better response to LAMA (e.g., tiotropium) add-ons; however, the proposed mechanism has yet to be elucidated, and this concept has, unfortunately, yet to be studied on a larger clinical scale.

Recent tiotropium trials have also demonstrated its safety and efficacy in paediatric populations. Phase II, randomized, dose-ranging studies of tiotropium Respimat (1.25 μg, 2.5 μg, and 5 μg for 4 weeks) in children aged 6–11 years [37] and adolescents aged 12–17 years [38] with symptomatic, moderate asthma despite maintenance treatment with ICSs demonstrated improvements in lung function (as indicated by mean peak FEV1); no dose-dependent response was found. In both studies, tiotropium Respimat was well tolerated with comparable adverse event rates across treatment and placebo groups [37, 38]. There are further five Phase III clinical trial studies in the paediatric population (Table 2 [20]), which are discussed in detail below.

The RubaTinA-asthma® study (NCT01257230) was conducted to assess the efficacy and safety of once-daily tiotropium added to ICSs (200–800 µg/day) with or without LTRAs in adolescent (12- to 17-year-old) patients with moderate persistent asthma [39]. At week 24, the improvement in peak FEV1 within 3 hours (FEV1_(0–3_ _h)_) was statistically significant with both tiotropium doses compared with placebo: 5 µg tiotropium, adjusted mean difference 174 mL (95% CI: 76, 272 mL; *p* < 0.001) and 2.5 µg tiotropium, 134 mL (95% CI: 34, 234 mL; *p* < 0.01) [39]. PensieTinA-asthma® was a double-blind, parallel-group trial (NCT01277523) assessing the effect of once-daily tiotropium Respimat add-on to ICSs (400–1600 µg/day) plus one or more controllers, or ICSs (200–800 µg/day) plus two or more controllers in adolescent (12- to 17-year-old) patients with severe persistent asthma [40]. In this study, tiotropium Respimat 5 µg provided numerical improvements in peak FEV1_(0–3_ _h)_ compared with placebo (90 mL; *p*=0.104); however, significant improvements were seen with the 2.5 µg dose (111 mL; *p*=0.046). While there were positive trends for improvements in lung function and asthma control, the primary efficacy endpoint was not met—this may have been related to the presence of a pronounced placebo response and the relatively short trial period (12 weeks); in comparison, the Phase III tiotropium studies of adolescents [39] and adults [23] with moderate asthma were analyzed at 24 weeks, while those of adults with severe asthma were analyzed at 48 weeks [22].

VivaTinA-asthma®, a 12-week, double-blind, placebo-controlled trial (NCT01634152) was the first Phase III study of tiotropium Respimat in children with severe symptomatic disease. It was conducted in children aged 6–11 years to assess the safety and efficacy of tiotropium Respimat add-on to high-dose ICSs (>400 µg/day) with one or more controller medications, or medium-dose ICSs (200–400 µg/day) with two or more controller medications in severe persistent asthma [41]. Compared with placebo, tiotropium 5 μg add-on therapy significantly improved the primary endpoint, peak FEV1_(0–3_ _h)_ (139 mL (95% CI: 75, 203; *p* < 0.001)). Once-daily tiotropium was also well tolerated as an add-on therapy to ICSs with other maintenance therapies in children with severe symptomatic asthma [41]. The objective of the CanoTinA-asthma® study (NCT01634139) was to examine the efficacy and safety of once-daily tiotropium Respimat as an add-on therapy in children (6–11 years old) with moderate persistent asthma on medium-dose ICSs (200–400 µg/day). Both doses of tiotropium Respimat, 5 µg and 2.5 µg, significantly improved FEV1_(0–3_ _h)_ at week 24, with adjusted mean differences of 164 mL and 170 mL versus placebo, respectively (*p* < 0.01). In children with moderate symptomatic asthma, once-daily tiotropium as an add-on to usual maintenance therapy was again considered safe and well tolerated [42].

Finally, NinoTinA-asthma® was the first study (NCT01634113) to evaluate the efficacy and safety of tiotropium in preschool children (1–5 years old) with persistent asthma on a stable dose of ICSs. This study found that tiotropium is a well-tolerated add-on option in this population and suggested that there may be a reduction in risk of asthma exacerbations, which were reported as adverse events in this trial [43]. While the current FDA approval of tiotropium Respimat in asthma applies to those aged 6 and older, data suggest that this device is easy to use by younger age groups as well. A handling study assessing the use of the Respimat inhaler in children aged 5 years and below concluded that it was suitable for children in this age group, although these younger patients are advised to add a valved holding chamber to facilitate its use [44]. To date, the studies in children and adolescents have only evaluated tiotropium as an add-on therapy to at least ICSs across a range of asthma severities; however, a comparative assessment of LABA versus LAMA add-on therapy in the paediatric population has yet to be published.

In clinical trials, the reported rates of discontinuation of tiotropium were similar to placebo arms and ranged from 1 to 11% [18, 22, 23, 45]. There are limited reported data on tiotropium compliance. In the TALC study, the compliance rate was 93.0% (similar to the salmeterol arm) [21], whereas in BELT—an open-label, randomized, pragmatic study which compared tiotropium versus salmeterol add-on therapy to ICSs in black adults with moderate-to-severe asthma for a mean follow-up period of 10 months—the compliance rate was 60% (also similar to the salmeterol arm) [46]. In a randomized, dose-ranging study evaluating tiotropium in symptomatic adolescents (aged 12–17), median compliance ranged from 85 to 87% across treatment groups [38].

Published prescribing information indicates that the most common adverse reactions of tiotropium (i.e., >5% incidence in the 1-year placebo-controlled trials) include upper respiratory tract infections, rhinitis and sinusitis, pharyngitis, dry mouth, nonspecific chest pain, dyspepsia, and urinary tract infections [47]. Tiotropium bromide is contraindicated in patients with a known hypersensitivity to it, atropine, or its derivatives (including ipratropium), and as per other anticholinergic medications, tiotropium should be used with caution in patients with narrow-angle glaucoma or urinary retention [48]. Tiotropium is predominantly renally excreted, and in patients with a creatinine clearance of <50 mL/min (i.e., moderate-to-severe renal impairment), it is to be used only if the anticipated benefit outweighs potential risk, and they should be monitored for the development of anticholinergic side effects [48].

In the COPD literature, there has been concern with regard to inhaled anticholinergics (including tiotropium) and their link to cardiovascular events and mortality [49], but subsequent data from the 4-year UPLIFT study did not support an association between tiotropium HandiHaler and risk of myocardial infarction, stroke, or death from cardiovascular causes [9]. The safety of tiotropium in the Respimat form in COPD has been debated: two systematic reviews and meta-analyses [50, 51] and a large cohort study [52] suggested that its use is associated with an increase in mortality of ∼30–50%, with the association being the strongest for cardiovascular and/or cerebrovascular death; however, a large randomized, double-blind parallel group trial of over 17,000 patients with COPD found that, during a mean follow-up of 2.3 years, tiotropium Respimat was noninferior to HandiHaler in the primary endpoint of risk of death and that causes of death and rates of major adverse cardiovascular events were similar across these devices [53].

The earlier-described Phase II and III trials of tiotropium in asthma have demonstrated a favourable adverse effect profile, with event rates that are comparable across different doses and placebo arms. However, in these trials, the safety and tolerance data resulted from analysis of secondary outcomes. In a randomized, double-blind, placebo-controlled parallel group trial of 285 patients with asthma by Ohta et al., tiotropium Respimat (at doses of 5 and 2.5 μg once daily) use over 52 weeks led to a similar rate of the primary outcome of adverse events (AEs) versus placebo (88.6% for 5 μg, 86.8% for 2.5 μg; 89.5% for placebo); the most commonly reported event in tiotropium Respimat 5 μg, 2.5 μg, and placebo groups, respectively, was “nasopharyngitis” (48.2, 44.7, and 42.1%), followed by “asthma” (28.9, 29.8, and 38.6%), “decreased PEFR” (15.8, 7.9, and 21.1%), “bronchitis” (9.6, 13.2, and 7.0%), “phayngitis” (7.9, 13.2, and 3.5%), and “gastroenteritis” (10.5, 3.5, and 5.3%) [54]. Pooled data from seven Phase II and III adult asthma trials (all randomized and double-blinded)—including the study by Ohta et al.—also found that the percentage of AEs was comparable between treatment tiotropium and placebo groups: the most frequent ones were “asthma,” “decreased PEFR,” and “nasopharyngitis”; the overall incidence of dry mouth and cardiac disorder AEs was comparable across all groups (<1% and <1.5%, resp.) [55].

Based on the established safety and efficacy data of tiotropium Respimat (Spiriva Respimat, Boehringer Ingelheim International GmbH), in September 2015, the FDA had approved its use in the U.S. as a long-term, once-daily maintenance therapy in asthma patients aged 12 and older; the approval was expanded to ages 6 and older in February 2017. The recommended dose in the U.S. is two inhalations of 1.25 μg once daily [56]. In Canada, tiotropium Respimat has been approved as an add-on maintenance bronchodilator in adult patients with asthma who continue to be symptomatic on a combination of ICSs (at minimum, equivalent to ≥500 μg fluticasone/day or ≥800 μg budesonide/day) plus LABAs and who have experienced ≥1 severe exacerbations in the previous year, at the recommended dose of two inhalations of 2.5 μg once daily, which parallels the approval in the European Union [48, 57]. It is unclear as to why these agencies have different approved doses; however, as reviewed earlier, both the 2.5 and 5 μg doses of tiotropium have been found to have comparable effects when studied together. Tiotropium has also been approved in Japan for patients aged 15 years and over, and it is undergoing review in other countries [58].

## 5. Further Perspectives

Recent studies have assessed the real-world effectiveness of tiotropium. A retrospective study of 2042 adults with asthma found that, in the first year following the addition of tiotropium (93% via HandiHaler, 7% via Respimat at 5 μg), there was a decrease in the incidence of both primary outcomes of exacerbations (patients experiencing ≥1 exacerbation were decreased from 37 to 27%, *p* < 0.001) and respiratory events requiring antibiotic prescriptions (from 58 to 47%, *p* < 0.001) [59]. Unlike the earlier-presented data from the Phase III randomized trials, no significant changes in lung function were found with the addition of tiotropium; this negative finding may be related to the inclusion of smokers (∼50% had an active smoking history, whereas those with >10 pack-year smoking history were excluded from trials) and the limited control on lung function data inherent to a retrospective study including timing of the measurements with regard to time of day, or relative to tiotropium initiation [59]. In a randomized, open-label, parallel-group pragmatic trial of 1070 black adults with moderate-to-severe asthma, the effect of tiotropium HandiHaler as add-on therapy to ICSs on time to first exacerbation was compared with LABAs (salmeterol or formoterol) [46]. Over a follow-up period of up to 18 months, the time to first exacerbation, as well as the mean number of exacerbations per person-year, did not differ between the tiotropium and LABA arms. There was also no difference in the two treatment groups in the secondary outcomes of FEV1, rescue medication use, and other patient-reported outcomes (AQLQ, ACQ, and Symptom Utility Index).

Cost-effectiveness represents an additional factor to consider when determining the real-world application of a medication. In 2014, Willson et al. found tiotropium to be a cost-effective add-on therapy, from the perspective of the UK National Health Service, to adult asthma patients uncontrolled on ICS-LABA combination by analyzing the PrimoTinA-asthma trial database [60]. Using a Markov modelling framework—which included seven mutually exclusive health states (three states of asthma control, three states of exacerbation, and death)—the authors analyzed costs and quality-adjusted life-years (QALYs). The model, utilizing the £20,000–£30,000 per QALY-gained willingness-to-pay threshold that is commonly accepted in the UK, found that the tiotropium add-on therapy generated an incremental 0.19 QALYs and £5389 in costs over a lifetime, resulting in an incremental cost-effectiveness ratio of £28,383 per QALY gained [60, 61]. Essentially, the addition of tiotropium, while increasing drug acquisition costs, was found to reduce exacerbation-related expenditures and also improve patients' quality of life.

The incorporation of tiotropium at Step 4 in recent iterations of the GINA guidelines targets the unmet need for optimal disease control in those with persistent, severe asthma, serving as a well-tolerated add-on therapy to ICS-LABA. However, given the results of director comparator studies between salmeterol and tiotropium as add-on therapies to ICSs, one wonders if LAMAs could serve as an equivalent alternative to LABAs in those with asthma and, as such, be positioned earlier in stepwise therapy. When compared to salmeterol, tiotropium was found to have similar efficacy in improving lung function (AM and PM PEF, FEV1) measured at 14 and 24 weeks in patients with moderate, persistent asthma with confirmed bronchodilator reversibility [21, 23]. In a pragmatic trial conducted over 18 months, tiotropium was found to have similar effects as LABA (salmeterol or formoterol) therapy on time to first exacerbation [46]. However, a Cochrane Library systematic review concluded that current evidence is not strong enough to support LAMAs as a LABA substitute, given that the comparative effects on exacerbations and serious adverse events are unclear based on short trial duration, as well as the larger evidence base supporting LABA versus placebo as an ICS add-on [26]. As such, the decision to favour LAMAs as an add-on versus LABAs would be on an individual patient level. Some studies, including the TALC study comparing tiotropium versus salmeterol, have shown that patients can develop tolerance to LABAs and impaired SABA response [21, 62–64], and thus, tachyphylaxis to LABAs could preferentially favour LAMAs as an ICS add-on therapy. Furthermore, follow-up analyses of the TALC study data found that adult asthma patients with higher cholinergic tone (as indicated by a lower resting heart rate) and the extent of airway obstruction (as indicated by the FEV1/FVC ratio) predicted a positive clinical response to tiotropium (versus salmeterol); ethnicity, gender, atopy, IgE levels, asthma duration, and BMI were not predictors [65]. While that study did not demonstrate sputum eosinophilia as a predictor of tiotropium response, sputum neutrophilia has been shown in another study to correlate positively with the FEV1 improvement at 4 weeks conferred by the addition of tiotropium [36]. It is hoped that future studies will assess these factors in a larger, prospective fashion to provide a practical and potentially guideline-based approach to long-acting bronchodilator choice in the clinical setting.

With regard to direct comparator trials, tiotropium as an add-on therapy to symptomatic asthma patients on at least ICSs has been demonstrated to be superior to doubling of ICSs [21], and comparable to LABA addition in adults [21, 23, 45, 46], across various clinical outcomes. However, the relative place and preference of tiotropium with respect to other potential GINA Step 4 strategies (e.g., theophylline and leukotriene receptor antagonists (LTRAs)) have yet to be addressed directly by large, blinded, randomized trials. Given the availability of other effective and safe treatments and the relatively modest properties on bronchodilation and symptom control [66–69], clinical practice guidelines have not recommended theophylline as a preferred controller therapy. However, its low cost offers an advantage over other add-on therapies added to ICSs, which may favour its use in developing nations [70]. There are controversies in the clinical evidence supporting the efficacy of LTRAs; for example, a meta-analysis of six clinical studies assessing montelukast as an add-on therapy in mild-to-moderate asthma showed significantly improved effectiveness in symptom control and exacerbation risk compared with ICSs [71]. In contrast, in other studies of patients treated with high-dose ICSs (≥1000 μg), the addition of montelukast showed no improvement in symptoms, lung function, or reduced SABA use [72, 73]. A Cochrane review published this year concluded that the addition of LTRAs reduces moderate and severe exacerbations and improves lung function and asthma control compared with the same dose of ICSs but that the evidence does not support this class of controller therapy as an ICS-sparing agent [74]. LTRAs may be useful in particular situations, including those intolerant of or unwilling to use ICSs and those with allergic rhinitis, aspirin-induced asthma, viral-induced wheezing, and asthma in the elderly [1, 75]. Data from a randomized, but unblinded, study of 297 patients on budesonide (400 μg/day) found that a 6-month trial of add-on montelukast safely improved FEV1, SGRQ, and rescue medication use at a faster rate than add-on doxofylline or tiotropium [76].

## 6. Summary

Tiotropium has recently been demonstrated as a well-tolerated and effective add-on treatment for adults, adolescents, and children with persistent, symptomatic asthma. The demonstrated efficacy and safety profiles, regardless of baseline characteristics and asthma severity, are encouraging. Furthermore, the effect of tiotropium on various clinical outcomes is consistent across patient subgroups and phenotypes which have historically imposed treatment challenges, including obesity, the elderly, and noneosinphilic asthma, which makes tiotropium a worthy consideration as an add-on bronchodilator in the treatment of asthma patients on, at minimum, inhaled corticosteroids. Future research is needed to investigate the long-term effectiveness of tiotropium, its relative efficacy, and safety compared to alternative treatment options at GINA Step 4 and its role as an alternative maintenance inhaler to LABAs.

## Figures and Tables

**Table 1:** Results of Phase III adult asthma studies with tiotropium Respimat 5 µg.

Study name	Treatment duration (weeks)	*N*	Primary and key secondary endpoints	Difference from placebo	
PrimoTinA-asthma severe persistent asthma [21]	48	912	Peak FEV1, week 24, mean (CI) mL	86 (20, 152) (*p*=0.01) trial 1	154 (91, 217) (*p* < 0.001) trial 2
			Trough FEV1, week 24, mean (CI) mL	88 (27, 149) mL (*p*=0.01) trial 1	111 (53, 169) mL (*p* < 0.001) trial 2
			Time to first severe exacerbation	21% reduction in risk (HR 0.79; 95% CI: 0.62, 1.00; *p*=0.03)	
			ACQ-7, adjusted mean score	NS	−0.2 (*p*=0.003) trial 2
MezzoTinA-asthma moderate persistent asthma [22]	24	2103	Peak FEV1, mean (CI) mL	198 (142, 253) mL (*p* < 0.0001) trial 1	169 (116, 222) mL (*p* < 0.0001) trial 2
			Peak FVC, mean (CI) mL	102 (42, 162) mL (*p*=0.0008) trial 1	89 (30, 147) mL (*p*=0.0031) trial 2
			ACQ-7, adjusted mean score	−0.12 (SD 0.04; *p*=0.0084)	
GraziaTinA-asthma mild persistent asthma [23]	12	465	Peak FEV1, week 12, mean (CI) mL	128 mL (95% CI: 57, 199; *p* < 0.001)	
			Trough FEV1, week 12, mean (CI) mL	122 mL (95% CI: 49, 194; *p* < 0.001)	
			ACQ-7 total score, week 12	0.014 (95% CI: −118, 0.146; *p*=0.83)	

ACQ-7, 7-question Asthma Control Questionnaire; CI, confidence interval; FEV1, forced expiratory volume in 1 second; FVC, forced vital capacity; HR, hazard ratio; ICSs, inhaled corticosteroids; NS, not significant; SD, standard deviation. Table reproduced from E. R. McIvor and R. A. McIvor [20], under the Creative Commons Attribution License/public domain.

**Table 2:** Phase III studies with tiotropium Respimat in children and adolescents with asthma.

Study name	Patients (asthma severity and age)	Treatment duration (weeks)	Baseline therapy	*N* (treatment group)	Study drug
RubaTinA-asthma	Moderate persistent 12- to 17-year-olds	48	At least ICSs	259	Tiotropium Respimat 2.5 and 5 µg
PensieTinA-asthma	Severe persistent 12- to 17-year-olds	12	ICSs + ≥1 controller	257	
CanoTinA-asthma	Moderate persistent 6- to 11-year-olds	48	At least ICSs	270	
VivaTinA-asthma	Severe persistent 6- to 11-year-olds	12	At least ICSs/+ ≥1 controller	262	
NinoTinA-asthma	Persistent 1- to 5-year-olds		At least ICSs	67	

ICSs, inhaled corticosteroids; LABA, long-acting β_2_-agonist. Table reproduced from E. R. McIvor and R. A. McIvor [20], under the Creative Commons Attribution License/public domain.
